# Innovative regenerative medicines in the EU: a better future in evidence?

**DOI:** 10.1186/s12916-017-0818-4

**Published:** 2017-03-08

**Authors:** Mark S. Corbett, Andrew Webster, Robert Hawkins, Nerys Woolacott

**Affiliations:** 1 Centre for Reviews and Dissemination, University of York, Heslington, York YO10 5DD UK; 2 Science and Technology Studies Unit, Department of Sociology, University of York, Heslington, York YO10 5DD UK; 3 Medical Oncology, The Christie Hospital and University of Manchester, Wilmslow Road, Manchester, M20 4BX UK

**Keywords:** Regenerative medicine, European Medicines Agency, Cell therapy, Gene therapy

## Abstract

**Background:**

Despite a steady stream of headlines suggesting they will transform the future of healthcare, high-tech regenerative medicines have, to date, been quite inaccessible to patients, with only eight having been granted an EU marketing licence in the last 7 years. Here, we outline some of the historical reasons for this paucity of licensed innovative regenerative medicines. We discuss the challenges to be overcome to expedite the development of this complex and rapidly changing area of medicine, together with possible reasons to be more optimistic for the future.

**Discussion:**

Several factors have contributed to the scarcity of cutting-edge regenerative medicines in clinical practice. These include the great expense and difficulties involved in planning how individual therapies will be developed, manufactured to commercial levels and ultimately successfully delivered to patients. Specific challenges also exist when evaluating the safety, efficacy and cost-effectiveness of these therapies. Furthermore, many treatments are used without a licence from the European Medicines Agency, under “Hospital Exemption” from the EC legislation. For products which are licensed, alternative financing approaches by healthcare providers may be needed, since many therapies will have significant up-front costs but uncertain benefits and harms in the long-term. However, increasing political interest and more flexible mechanisms for licensing and financing of therapies are now evident; these could be key to the future growth and development of regenerative medicine in clinical practice.

**Conclusions:**

Recent developments in regulatory processes, coupled with increasing political interest, may offer some hope for improvements to the long and often difficult routes from laboratory to marketplace for leading-edge cell or tissue therapies. Collaboration between publicly-funded researchers and the pharmaceutical industry could be key to the future development of regenerative medicine in clinical practice; such collaborations might also offer a possible antidote to the innovation crisis in the pharmaceutical industry.

## Background

“*Cure for blindness found*” proclaimed a front page headline of a UK newspaper [1]. The article’s text revealed a different story, of a promising line of research based on clinical trial data from just a single patient treated with an embryonic stem cell therapy, for which it “*will be some months before the full impact of it* [the treatment] *on her sight is known*”. Although the fullness of time may yet reveal this new treatment to be a cure for blindness, this example illustrates the weight of expectation often placed on innovative new ‘regenerative medicines’ to transform the future of healthcare. Regenerative medicine – which is not a new field of medicine as it encompasses bone marrow or organ transplants – deals with the process of replacing or regenerating human cells, tissues or organs to restore or establish normal function [2]. Most new regenerative medicines are classed by the European Medicines Agency (EMA) as being ‘advanced-therapy medicinal products’ (ATMPs), which are engineered regenerative medicines encompassing cell-based therapies (often using stem cells or progenitor cells to produce tissues), gene therapies and tissue-engineered therapies.

The Committee for Advanced Therapies is the EMA committee responsible for assessing the quality, safety and efficacy of ATMPs (and for following scientific developments in the field). Several EMA regulatory pathways exist to facilitate accelerated access to treatments where there is an unmet patient need. These include approval under exceptional circumstances (1993), conditional marketing authorisation (2005), accelerated assessment (2005), parallel scientific advice between EMA and Food and Drug Administration (FDA; 2009), and the adaptive licensing pilot programme (2014). Nevertheless, despite 318 relevant trials having been performed in Europe between 2004 and 2010, [3] only eight ATMPs have been granted a marketing licence by the EMA, namely ChondroCelect (2009), Glybera (2012), MACI (2013), Provenge (2013), Holoclar (2015), Imlygic (2015), Strimvelis (2016), and Zalmoxis (2016). The main sponsors were academic organisations, charities and small companies, all of which are stakeholders who tend to have limited resources with regard to both financing and the capacity to navigate the required regulatory procedures [3]. This mismatch between the number of promising ideas and translation to actual patient benefit [4] may partly be due to key differences between regenerative medicines and conventional pharmaceuticals. Regenerative medicines are often truly personalised and therefore expensive and difficult to manufacture; evaluation of their efficacy, safety and cost-effectiveness may also be challenging [5, 6].

Nevertheless, there appears a new sense of urgency to address these issues. In the UK, a House of Lords Science and Technology Committee inquiry into regenerative medicine identified barriers to translation and commercialisation and recommended solutions. In response to its findings – published in 2013 [7] – the National Institute for Health and Care Excellence (NICE) was asked to commission a study to assess whether its current appraisal methods and processes were appropriate to evaluate regenerative medicines. We were part of the team that performed that study, which included an assessment of previous evaluations of regenerative medicines by NICE, the EMA and the FDA [8, 9]. More recently (in April 2016), the House of Commons Science and Technology Committee announced it was undertaking an inquiry into regenerative medicine, which will report in early February 2017. In this article, we discuss some of the historical causes for the scarcity of licensed innovative regenerative medicines, together with possible reasons to be more optimistic for the future. This paper arose as a result of our work on the aforementioned study for NICE, which was commissioned by the Regenerative Medicine Expert Group.

## Discussion

### The evidence-access trade-off

Although the randomised controlled trial is the expected level of evidence for regulatory assessments, it is likely that many evidence submissions for regenerative medicines will comprise small, single-arm, short-term, early phase clinical trials. These sub-optimal study designs are likely to produce biased and imprecise results. However, the use of such designs is not inevitable and is unlikely to be through choice, but is instead a consequence of the type of patients targeted by new therapies. Populations with rare, severe or advanced disease may be very small, and therefore adequate recruitment to trials with two treatment arms would require many centres, much time and great expense. Further, where no alternative treatments exist, and patients have life-threatening or severe disease, randomisation to a control group is likely to be ethically unacceptable or problematic, as may the requirement for lengthy follow-up before licence submission. This is particularly likely to be a problem when initial studies on small numbers of patients have shown spectacular results [10]. Trials utilising alternative approaches to conventional randomisation might also be considered when rare diseases are studied [11]. For example, responsive-adaptive randomisation maximises allocation to the most effective treatment and minimises the required sample size. Such a ‘play the winner’ design has the potential to reduce the number of patients allocated to less effective treatment, therefore reducing the ethical concerns associated with randomisation, though it is limited to studies that assess rapidly available outcomes. However, for new types of study design such as this, the magnitude of the risks of bias are not yet well understood [12]; in reality, single-arm studies are therefore most likely to be submitted for licensing purposes.

Another issue often encountered is the use of surrogate endpoints – laboratory or physiological measures used to predict or provide an early measure of therapeutic effect – rather than real clinical (patient-important) endpoints. Surrogate endpoint data are quicker and easier to acquire than real clinical endpoint data, thus saving valuable time in the licensing process (for both manufacturers and patients). However, there is often considerable uncertainty about the strength of the relationship between a given surrogate and its relevant real clinical endpoint; this is problematic because trial results based on surrogate endpoints are not likely to be reliable if the surrogate has not been properly validated. It is therefore recommended that, before a surrogate outcome is accepted, a systematic review should be conducted examining the evidence for the validation of the surrogate-final outcome relationship. To validate a surrogate endpoint such reviews must demonstrate that there is adequate evidence from several sources, including clinical trials, epidemiological/observational studies and pathophysiological studies (of biological plausibility of the relationship) [13].

Additionally, for treatments studied in highly specialised settings, scepticism about results may arise from evidence suggesting that single-centre trials tend to produce significantly larger effect estimates than multi-centre trials. A systematic review of 82 critical care medicine randomised controlled trials found significantly larger treatment effects for all-cause mortality in single-centre trials when compared with multi-centre trials (ratio of odds ratios, 0.64; 95% CI, 0.47–0.87) [14]. Where ATMPs are likely to be delivered from more than just a single clinic site, efficacy results from single-centre trials should be considered cautiously by decision makers.

The implications of such evidential problems will largely depend on both the level of unmet need in the studied population and the likeliness of cure or improvement without experimental treatment. This can be illustrated by comparing two quite recently licensed therapies that claimed to meet unmet patient needs: Holoclar (a stem cell-based therapy for treating corneal lesions resulting from burns to the eye) and Glybera (a gene therapy for treating familial lipoprotein lipase deficiency with associated pancreatitis). It seems Holoclar and Glybera had very different approval histories, primarily due to differences in the likelihood of patient improvement without new therapy. Despite the clinical evidence for Holoclar being based on uncontrolled retrospective data, the results (approximately half the patients had improved visual acuity) were still sufficiently impressive for the EMA to grant a conditional licence on the first application. The prospective confirmatory study required as part of the EMA’s conditional approval should clarify Holoclar’s efficacy and safety.

The application for Glybera was also supported by single-arm trial evidence. However, while the evidence for Holoclar was deemed sufficiently robust, for Glybera, there were concerns that the apparent treatment benefit may have been due to chance, resulting in Glybera’s long and protracted route to marketing authorisation. Negative committee opinions were issued in June 2011, with approval finally granted in July 2012 with a more restrictive licence than was originally applied for. In 2015, the manufacturer of Glybera dropped plans to seek approval for the therapy in the US following the FDA’s request for further clinical data.

As outlined earlier, the EMA has several regulatory pathways that attempt to create a balance between shorter approval times for promising medicines for populations with high unmet medical needs, and ensuring a flow of evolving satisfactory information on efficacy and safety. The most recent update – the adaptive pathways pilot programme – utilises existing regulatory processes, and is a prospectively planned adaptive approach to bringing treatments to market with an initial focus on patients with a high unmet need. It will be more of a staggered, iterative system than previous approval pathways. Such a ‘life-cycle approach’ to acquiring and (re)assessing evidence will consider the basis of decision-making in the stages of a product’s life cycle, namely development, licensing, reimbursement, monitoring/post-licence evidence and utilisation [15, 16].

Improvements to this life-span approach are developing at pace. For example, with Medicine Adaptive Pathways to Patients the development plan across target populations and indications will be agreed up-front with the EMA. Plans may include a range of studies, such as randomised controlled trials, single-arm studies, pragmatic trials and other forms of real world study [17]. A newly formed public–private project called ADAPT SMART (Accelerated Development of Appropriate Patient Therapies: a Sustainable, Multi-stakeholder Approach from Research to Treatment-outcomes), funded by the EU Innovative Medicines Initiative, focuses on laying the foundations for Medicine Adaptive Pathways to Patients to be put into practice in Europe. The challenge for ADAPT SMART is to develop an approach to adaptive pathways that aligns the needs of all stakeholders, including patients, member state payers, regulators, medical practitioners and the industry [18]. Finally, the recent proposals announced by the UK Department of Health (2016) [19] to develop a more enabling environment for ‘strategically important transformative products’ are regarded as an additional vehicle through which ATMPs might be fostered. Crucial to this will be the establishing of ‘accelerated access partnerships’ between public and private sectors and the NHS of a form not seen before, suggesting that its success will depend as much on identifying transformative processes as it will on products [19].

### Development of ideas and scale-up to commercialisation

Most new regenerative medicines are developed by academic research institutions or small- and medium-sized enterprises. Ideas for new therapies are not uncommon, but it is difficult for new centres to enter the field under existing regulations; producing regenerative medicines in accordance with good manufacturing practices (to ensure quality, safety and efficacy) is expensive, and the ongoing costs are frequently overlooked by academic centres with no history of cell therapy manufacture. Successful academic centres are often those with pre-existing quality management systems and staff experienced in manufacturing more conventional cell therapy products (e.g. those relating to haematopoietic stem cell transplantation and lymphocyte infusion) [20].

The wide variation evident across the types of new cell-based medicines [21] highlights the importance of careful consideration of how individual therapies will be developed, manufactured and ultimately successfully delivered to patients in clinical practice settings to commercially viable levels. Key differences in issues will also arise depending on which of the two main types of cell is being used when developing a new therapy: autologous (bespoke) cell therapies, which are derived from an individual patient’s own cells and allogeneic (universal) cell therapies which are derived from a donor. A clear understanding of what will be needed for scale-up to commercial levels is particularly important. Autologous therapies have advantages over allogeneic therapies in terms of smaller start-up costs, simpler regulations, the potential for point-of-care processing, and ease of obtaining cells (in terms of time and resources). Allogeneic therapies have advantages over autologous therapies in terms of patient throughput, product consistency, quality control being applied to larger lots, and treatment delays from processing failures [22]. Of the eight ATMPs licensed to date, five have been autologous, two have been viral gene therapies (Glybera and Imlygic) and one allogeneic (Zalmoxis).

The Cell and Gene Therapy Catapult is an organisation dedicated to growing the UK cell and gene therapy industry by bridging the gap between academic research and full scale commercialisation. It promotes and develops the existing early phase manufacturing network; the UK’s small-scale academic facilities are a good source of materials for early-stage clinical trials although they are expected to reach full capacity within a few years as the industry matures. With this in mind, the Catapults’ work will be augmented by a £55 m large-scale manufacturing facility (due to open in 2017) [23]. The centre aims to provide an infrastructure to enable the manufacture of allogeneic or autologous cell therapies for later phase (II or III) clinical trials and commercial scale-up. For developing businesses, this will mean that finances need not be committed to a permanent commercial facility before it is known whether products are going to be both clinically useful and economically viable. The vision is that successful products will eventually be manufactured from purpose-built facilities operated by successful firms. Input from organisations such as the Cell and Gene Therapy Catapult could be crucial since company size appears to be an independent predictor of outcome of a marketing authorization application to the EMA – the smaller the company, the more likely a negative outcome. Direct interaction with regulators also appears to be a key predictor of success [24].

### Reimbursement by healthcare systems and evaluation of cost-effectiveness

How should we value and price therapies which might cure chronic or fatal diseases? How should we pay for them? Claims of long-term benefits (perhaps even cures), long-term safety issues due to persistence of therapeutic cells, and significant up-front costs are issues which raise particular challenges in the assessment of the cost-effectiveness of regenerative medicines. Even where there may be significant potential benefits over current therapies, these may not be known with a high level of certainty at the time of licensing [8, 9]. Furthermore, a key difference between regenerative medicines and conventional medicines is the possibility that therapies may change over time. For example, when the ATMPs MACI and ChondroCelect (treatments for knee cartilage defects) were assessed by NICE they were third generation products. This may pose further uncertainty problems since by the time long-term trial results become available, the particular studied therapy may well have been superseded by a (apparently superior) next-generation treatment.

For EMA licensing purposes, a sponsor must demonstrate a favourable benefit-risk balance. However, the level of evidence required to achieve this can be less than that needed to estimate the relative effectiveness compared to current practice, or to quantify long-term treatment benefits. Since this latter information is essential for reliable assessment of cost-effectiveness, developers may find it is more difficult to demonstrate cost-effectiveness for reimbursement than it is to demonstrate efficacy for licensing. Schemes that allow development of further evidence or entail a risk-sharing component (between the payer and the manufacturer) may therefore be required.

Managed entry agreements or performance-based risk sharing agreements (PBRSAs) are an increasingly common policy response for dealing with evidence base uncertainty. PBRSAs involve the performance of treatments being tracked in a defined patient population over a specified time period, with the level or continuation of reimbursement based on the health and economic outcomes achieved. PBRSAs fall under a variety of names and categories such as outcomes-based schemes, risk-sharing agreements, coverage with evidence development, access with evidence development, conditional licensing and managed entry schemes. Patient access schemes may also sometimes be linked to performance. There has always been much uncertainty about the ultimate real-world clinical and economic performance of new products; PBRSAs represent one mechanism for reducing this uncertainty [25].

Concern surrounding the potential high up-front costs of regenerative medicines and their affordability to healthcare systems means that alternative financing approaches may also have to be considered. These include pay for performance, where the total price is more directly related to therapy performance in clinical practice, and amortisation, where payments are spread over the expected duration of benefits [26]. The appropriateness of employing different discount rates and/or different rates over time is also an area requiring careful consideration, particularly for potentially curative therapies.

Successful adoption of newly licensed ATMPs may well depend on how they relate to existing clinical interventions. The manufacturer of ChrondroCelect – a licensed treatment for knee cartilage defects – recently announced the initiation of the withdrawal of marketing authorisation due to commercial reasons. The EMA’s marketing authorisation for MACI (also a therapy for knee cartilage defects) was suspended in September 2014 as an authorised manufacturing site no longer existed (the developer closed the site). A key issue here could be the availability of alternative, more cost-effective treatments; indeed, established treatments such as microfracture surgery have long been available for repairing knee cartilage defects. More recently, in December 2016, the FDA gave marketing authorisation to MACI in the USA, and Vericel will now try to build a new market for it there. ATMPs are likely to be expensive and these examples suggest that they may be most likely to succeed commercially where there is an unmet medical need.

### Remaining hurdles and uncertainties

Despite reasons to be optimistic about the future of regenerative medicine, further challenges abound. An important issue is that many therapies are currently used without a licence from the EMA under “Hospital Exemption” from the EC legislation (or via the “Specials” scheme in the UK). Such treatments are prepared on a non-routine basis according to specific quality standards and are used for individual patients in a hospital under the professional responsibility of a medical practitioner. The problem is that hospital exemptions are regulated at the national level, with different countries interpreting the legislation in different ways; harmonisation and clarity are needed in defining when treatments qualify. There is concern about the risk that too broad a use of hospital exemptions may deter the submission of marketing authorisation applications to the EMA [27].

Careful consideration of longer-term adverse effect profiles is also important, as they may not be straightforward. While many harms associated with pharmaceuticals may improve following discontinuation, for regenerative medicines, there is the possibility of prolonged harm, especially where cells persist long-term. Developing effective methods for inducing immune tolerance of allogeneic therapies also remains a challenge. Patients receiving allogeneic cells may need long-term immune suppression to avoid rejection. More broadly, concerns have been raised that the evidence for benefits to patients of adaptive pathway approaches is lacking or contradictory [28]. There is also concern about the follow-up evidence for some treatments granted conditional approval by the EMA, with inconsistencies and delays in the fulfilment of specific obligations [29, 30].

The optimum approach for manufacturing autologous therapies is likely to be difficult to predict. Autologous therapies can be manufactured centrally, although an example of the type of difficulties encountered with some centralised production models is provided by considering Provenge (sipuleucel-T), a cell-based immunotherapy for the treatment of prostate cancer. The process involved patient cells being cold-shipped to a manufacturing site, where target immune cells were isolated and activated. These were then cold-shipped back to the patient, re-infused and the process repeated three times. The product handling and manipulation was mostly manual, which led to high product operating costs. Although efforts were made to reduce costs by automating some process stages, this example highlights the importance of considering functionally closed and automated scale-out processes early in clinical development [31]. In May 2015, the EU marketing authorisation for Provenge was withdrawn at the request of the manufacturer for commercial reasons.

An alternative approach to producing autologous therapies centrally is scaling-out, rather than scaling-up (in a large facility). Historical successful examples of the creative use of existing multiple centres with technically-advanced facilities include organ, bone-marrow and stem-cell transplants [32]. However, achieving a high level of product quality with decentralised models requires highly standardised, robust and transparent manufacturing processes and platforms [33]. In-hospital micro-factories are also prominent, particularly for autologous procedures that entail significant surgery/patient contact; current examples include limbal stem cell transplantation and the bioengineered trachea. Nevertheless, whether multiple hospitals will be willing or able to commit to good manufacturing practice under licence is untested. The UK move towards ‘Cell and Gene Therapy Treatment Centres’ as recommended by the Advanced Therapies Manufacturing Taskforce (2016) [34] poses new challenges for hospitals and the clinical science system more generally in designing new treatment infrastructures – with specific skills sets, logistical and equipment demands, and regulatory oversight – for ATMPs. Centralised production of autologous therapies may be seen as more appropriate, as is currently happening with a therapy (CTL019) being developed by Novartis; CTL019 is one of a number of chimeric antigen receptor (CAR) T-cell blood cancer therapies.

Providing a good illustration of many of the issues raised in this article, CAR T-cell therapies are a regenerative medicine to watch out for in the near future. They may offer a potential cure for very ill patients with high unmet medical needs – typically patients with refractory/relapsed leukaemia – though they have potentially serious adverse effects. In autologous CAR T-cell therapies a patient’s T-cells are genetically modified, whereby the activated T-cells can attack and destroy leukaemia B-cells. These therapies have been under development for approximately 20 years, they are truly innovative and they have received much press attention due to very encouraging early phase trial results [8]; consequently, the use of a randomised controlled design in further trials would not be ethical in the patient populations being studied.

CAR T-cells are costly and complex to produce. For Novartis’s CTL019 the initial work was carried out in an academic setting with the treatments now being produced in centralised large scale facilities in preparation for licensing trials and marketing authorisation. Interestingly, in terms of the viability and cost-effectiveness of manufacture, CTL019 is being produced in the same facility as (the aforementioned) Provenge was. However, there appear to be key differences between these therapies: the benefits from CTL019 seem likely to be much greater than those from Provenge and CTL019 is frozen-shipped, so transportation and timing issues should be minimised. Novartis bought the facility from Dendreon, the biotech company that manufactured Provenge.

The CAR T-cell example also highlights the importance of adequately robust research both for marketing authorisation and beyond. When to treat with CAR T-cells, what pre-conditioning is needed, and how to manage toxicity due to cell persistence are just some of the issues which will need resolving.

**Fig. 1:**
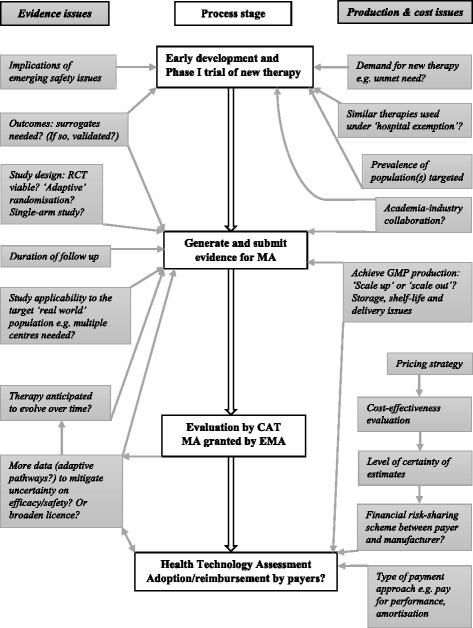
Overview of key stages and associated issues when bringing an advanced-therapy medicinal product to market in the EU

For future ATMPs, the importance and impact of the many issues and possible solutions discussed here will vary according to the specific therapy being developed. An overview is presented in Fig. 1.

## Conclusions

Despite the challenges involved in taking an ATMP from laboratory to marketplace, the EMA’s approval of Strimvelis and conditional approval of Holoclar provide recent examples of successful collaboration between publicly-funded researchers and the pharmaceutical industry [35, 36]. Such collaborations could be the antidote to the innovation crisis in the pharmaceutical industry, where much research is aimed at developing safe-bet ‘me too’ drugs which offer little or no benefit over treatments already available [37]. Collaboration may allow closer attention to the patient pathway and reduce time to market by ensuring more straightforward adoption into clinical practice [38].

The more flexible regulatory landscape, more appropriate range of options for reimbursement and increasing political interest and support structures do suggest a brighter future for regenerative medicine – the licensing of four ATMPs between 2015 and 2016 attest to this. Nevertheless, only time will tell as to whether future ‘cure for blindness’ headlines reflect the probable, rather than the possible.

## Abbreviations

ADAPT SMART: Accelerated Development of Appropriate Patient Therapies: a Sustainable, Multi-stakeholder Approach from Research to Treatment-outcomes
ATMP: Advanced-therapy medicinal product
CAR: Chimeric antigen receptor
EMA: European Medicines Agency
FDA: Food and Drug Administration
NICE: National Institute for Health and Care Excellence
PBRSAs: Performance-based risk sharing agreements
